# Cross-reactivity trends when selecting scFv antibodies against snake toxins using a phage display-based cross-panning strategy

**DOI:** 10.1038/s41598-023-37056-6

**Published:** 2023-06-22

**Authors:** Christoffer V. Sørensen, Line Ledsgaard, Helen H. K. Wildenauer, Camilla H. Dahl, Tasja W. Ebersole, Markus-Frederik Bohn, Anne Ljungars, Timothy P. Jenkins, Andreas H. Laustsen

**Affiliations:** Department of Biotechnology and Biomedicine, Technical University of Denmark, 2800 Kongens Lyngby, Denmark

**Keywords:** Drug discovery, Biologics, Antibody therapy, Biotechnology, Biologics, Antibody therapy

## Abstract

Antibodies with cross-reactive binding and broad toxin-neutralizing capabilities are advantageous for treating indications such as infectious diseases and animal envenomings. Such antibodies have been successfully selected against closely related antigens using phage display technology. However, the mechanisms driving antibody cross-reactivity typically remain to be elucidated. Therefore, we sought to explore how a previously reported phage display-based cross-panning strategy drives the selection of cross-reactive antibodies using seven different snake toxins belonging to three protein (sub-)families: phospholipases A_2_, long-chain α-neurotoxins, and short-chain α-neurotoxins. We showcase how cross-panning can increase the chances of discovering cross-reactive single-chain variable fragments (scFvs) from phage display campaigns. Further, we find that the feasibility of discovering cross-reactive antibodies using cross-panning cannot easily be predicted by analyzing the sequence, structural, or surface similarity of the antigens alone. However, when antigens share the (exact) same functions, this seems to increase the chances of selecting cross-reactive antibodies, which may possibly be due to the existence of structurally similar motifs on the antigens.

## Introduction

Antibodies have become a highly successful group of therapeutic molecules in recent decades due to their ability to bind antigens with high selectivity and specificity^1,2^. Additionally, antibodies can be developed to be cross-reactive and have broad toxin-neutralizing capabilities^3–5^, given a proper discovery strategy. These traits are especially relevant for developing therapies against indications such as infectious diseases and envenomings. However, cross-reactivity towards protein isoforms from different animal species is also of particular relevance for translational aspects between preclinical models and the clinical setting. This is especially important for diseases with endogenous targets, such as autoimmune diseases and cancer. For the discovery of cross-reactive antibodies, phage display is a key technology^6,7^, which can be applied together with specific methods such as cross-panning^3,8,9^, and/or next-generation sequencing analysis of parallel phage display pannings^10^. In this study, we explored the use of antibody phage display technology to isolate cross-reactive single-chain variable fragment (scFv) antibodies. As an in vitro antibody discovery method, phage display selection allows for a high level of control during the discovery process, such as the possibility to alter pH, alternate antigens, and reduce the antigen concentration, to mention a few^6,11^. Capitalizing on the ability to alternate antigens, we carried out several cross-panning strategies to enrich for cross-reactive binders. The antigens we aimed to discover cross-reactive antibodies against were toxins from venomous snakes. Snake toxins are a relevant group of proteins to use in this regard, as the discovery of cross-reactive (and broadly toxin-neutralizing) antibodies could potentially help save some of the approximately 100,000 lives that are lost to snakebites each year^12–14^.

**Figure 1:**
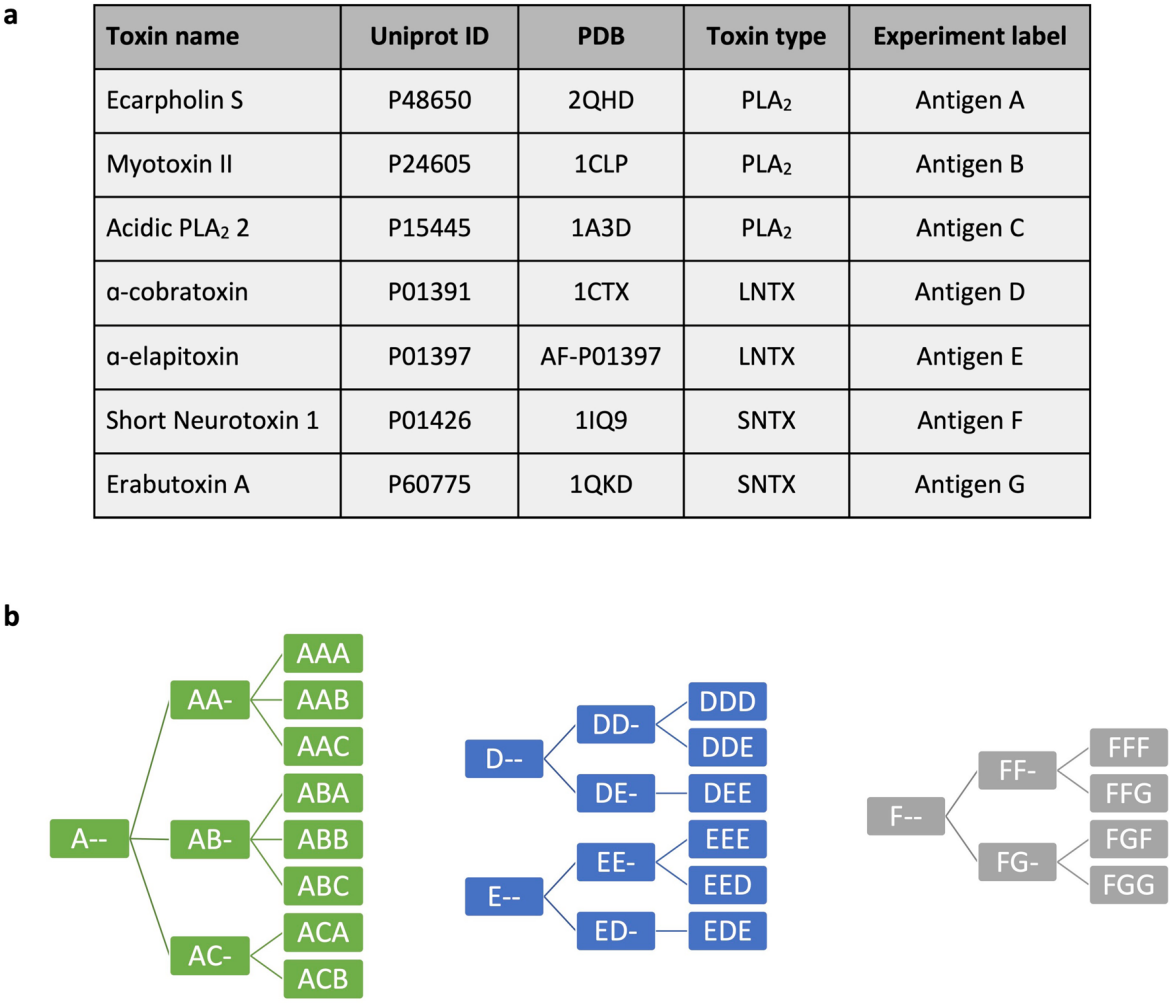
Overview of antigens and panning strategies used in this study. (**a**) 7 different snake toxins were used as antigens in this study divided into three different toxin groups: PLA_2_ = Phospholipase A_2_, LNTX = Long-chain α-neurotoxin, SNTX = Short-chain α-neurotoxin. (**b**) Three consecutive rounds of panning were performed. Letters refer to the antigen used in the respective panning round, i.e. A = Antigen A. Hyphens (-) denote that this round has not been carried out, i.e. A- = first panning round using antigen A. Green: PLA_2_ campaign, blue: LNTX campaign, grey: SNTX campaign.

Here, we present the comprehensive results from phage display selection campaigns using cross-panning strategies against three different groups of snake toxins (Fig. 1): three phospholipase A_2_s (PLA_2_s), two long-chain α-neurotoxins (LNTXs), and two short-chain α-neurotoxins (SNTXs). These campaigns were carried out across a range of antigens with different levels of similarity and show that in cases of low antigen similarity, the chance of discovering cross-reactive antibodies becomes low. Furthermore, this study demonstrates that implementing cross-panning strategies in antibody phage display selection campaigns can result in an increased fraction of cross-reactive scFvs in the panning outputs compared to selection campaigns using only a single antigen. Taken together, this study indicates that cross-panning may often be of utility when employing phage display technology to discover cross-reactive antibodies.

## Results

### Sequence, structural, and surface comparison of antigens

**Figure 2:**
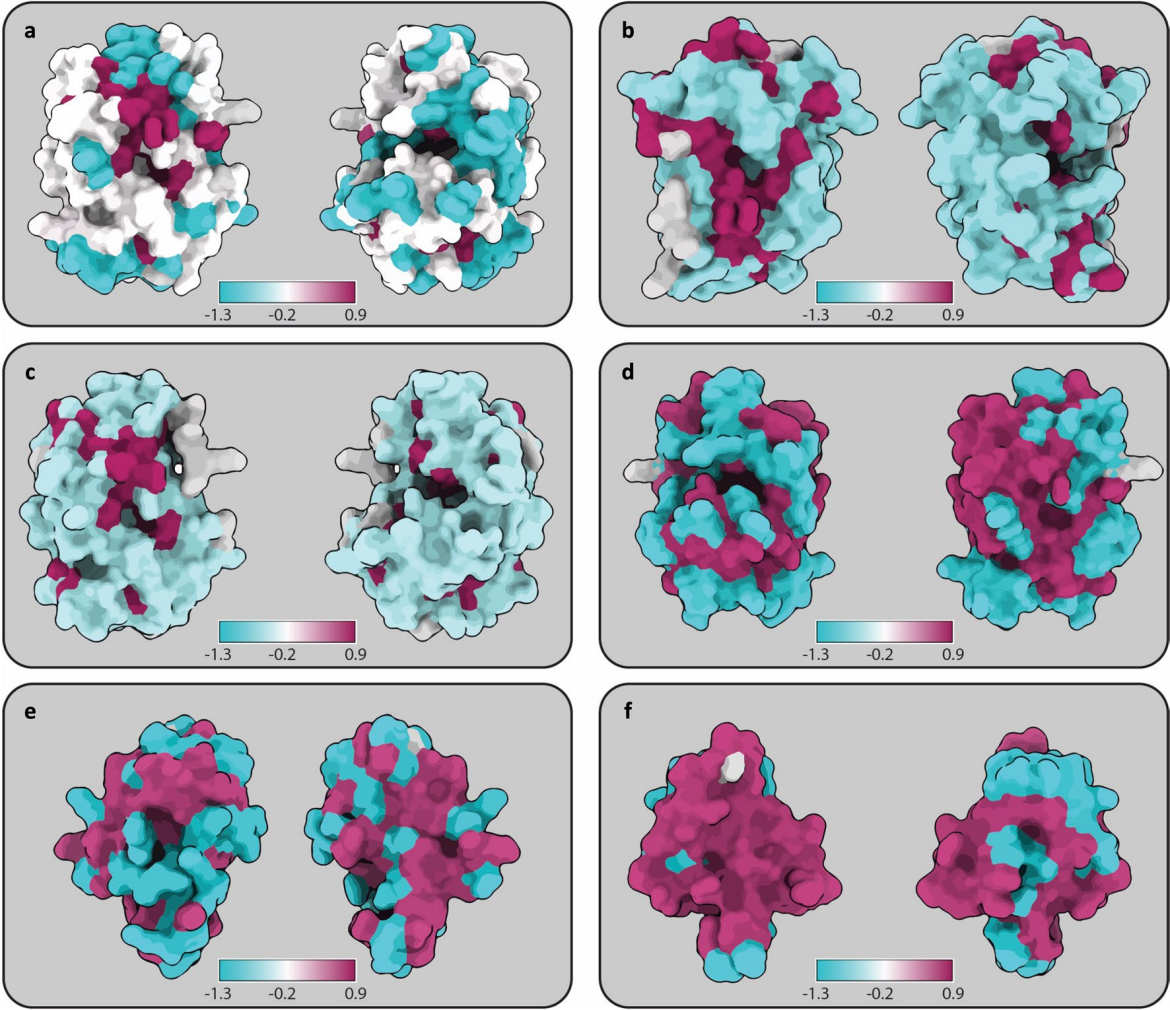
Visualization of antigen surface conservation. (**a**) Antigen A, B, and C. (**b**) Antigen B and C. (**c**) Antigen A and C. (**d**) Antigen A and B. (**e**) Antigen D and E. (**f**) Antigen F and G. Maroon indicates high and blue low conservation, with white representing elements present in one, but not the other. Conservation is calculated using the entropy-based measure from AL2CO and presented as log values^21^.

Prior to initiating the antibody discovery campaigns, a computational analysis of the similarity between the included antigens was performed. This was carried out using linear sequence similarity (Table 1), structural similarity (Table 1), and visual representation of surface conservation between the antigens (Fig. 2). The three phage display campaigns include toxins with a wide range of linear similarities ranging from 26% between the three PLA_2_s to 73% between the two more conserved SNTXs (Table 1). When comparing the structural similarity (RMSD scores), the SNTXs were again more conserved with pruned/unpruned RMSD scores of 0.58 Å/1.26 Å compared to pruned/unpruned scores of 0.73 Å/4.26 Å, 0.74 Å/4.52 Å, and 0.69 Å/1.42 Å for the PLA_2_s and 1.15 Å/3.06 Å for the LNTXs.

**Table 1:** A comparison of antigen similarity. Linear identity was carried out by aligning using CLC Main Workbench 21.0.4 with gap open cost and gap extension cost of 10. Upon superimposition of the protein structures by first creating a pairwise sequence alignment and then fitting the aligned residue pairs, the root-mean-square deviation (RMSD) was calculated. The default RMSD is measured across the whole protein alignment, whereas the pruned RMSD values only refer to sections successfully superimposed.

Antigens	Phage display campaign	Linear identity	RMSD between superimposed proteins (pruned)	RMSD between superimposed proteins (default)
Antigen A + B + C	PLA_2_	26%	Not applicable	Not applicable
Antigen A + C	PLA_2_	29%	0.73 Å	4.26 Å
Antigen B + C	PLA_2_	37%	0.74 Å	4.52 Å
Antigen A + B	PLA_2_	58%	0.69 Å	1.42 Å
Antigen D + E	LNTX	64%	1.15 Å	3.06 Å
Antigen F + G	SNTX	73%	0.58 Å	1.26 Å

A visual analysis of surface conservation among the antigens indicates that, collectively, the three PLA_2_s share only a limited number of surface regions (Fig. 2a). However, in a pairwise comparison between the PLA_2_s, it becomes apparent that antigen A and B share a large number of surface regions (Fig. 2d), while antigen C shows a relatively low surface similarity with both A and B (Fig. 2b and c). In the comparison of LNTXs (Fig. 2e), the level of surface conservation appears similar to that observed between the two most similar PLA_2_s (Fig. 2d). On the other hand, when instead comparing the SNTXs, there is a strikingly high degree of surface conservation (Fig. 2f).

### Phage ELISA screening of polyclonal phage outputs

Following three rounds of antibody phage display selection using cross-panning strategies with different combinations of antigens (Fig. 1), the phage outputs were analyzed using polyclonal phage ELISAs (Supplementary Fig. S1). These ELISAs showed enrichment of scFv-displaying phages not only against the antigens employed in the respective phage display campaign, but also against antigens not included in the panning process. For example, the phage pools after selection strategy AA-, AAA, and AAB, which were panned only against antigen A or a combination of antigen A and antigen B, also show binding to antigen C (Supplementary Fig. S1a). For the LNTX and SNTX campaigns (Supplementary Fig. S1b and c) the polyclonal scFv-displaying phages primarily bind the target antigens, and not the negative controls. However, for the PLA_2_ campaign, a few panning outputs showed accumulation of unspecific binders as well. Using the data from the polyclonal phage ELISAs, scFv genes were subcloned from selection rounds 2 and 3 into an scFv bacterial expression vector^15^. Soluble scFvs were then screened to include only the most promising ones for further characterization.

### Cross-panning increased the percentage of cross-reactive scFvs from two out of three panning campaigns

**Figure 3:**
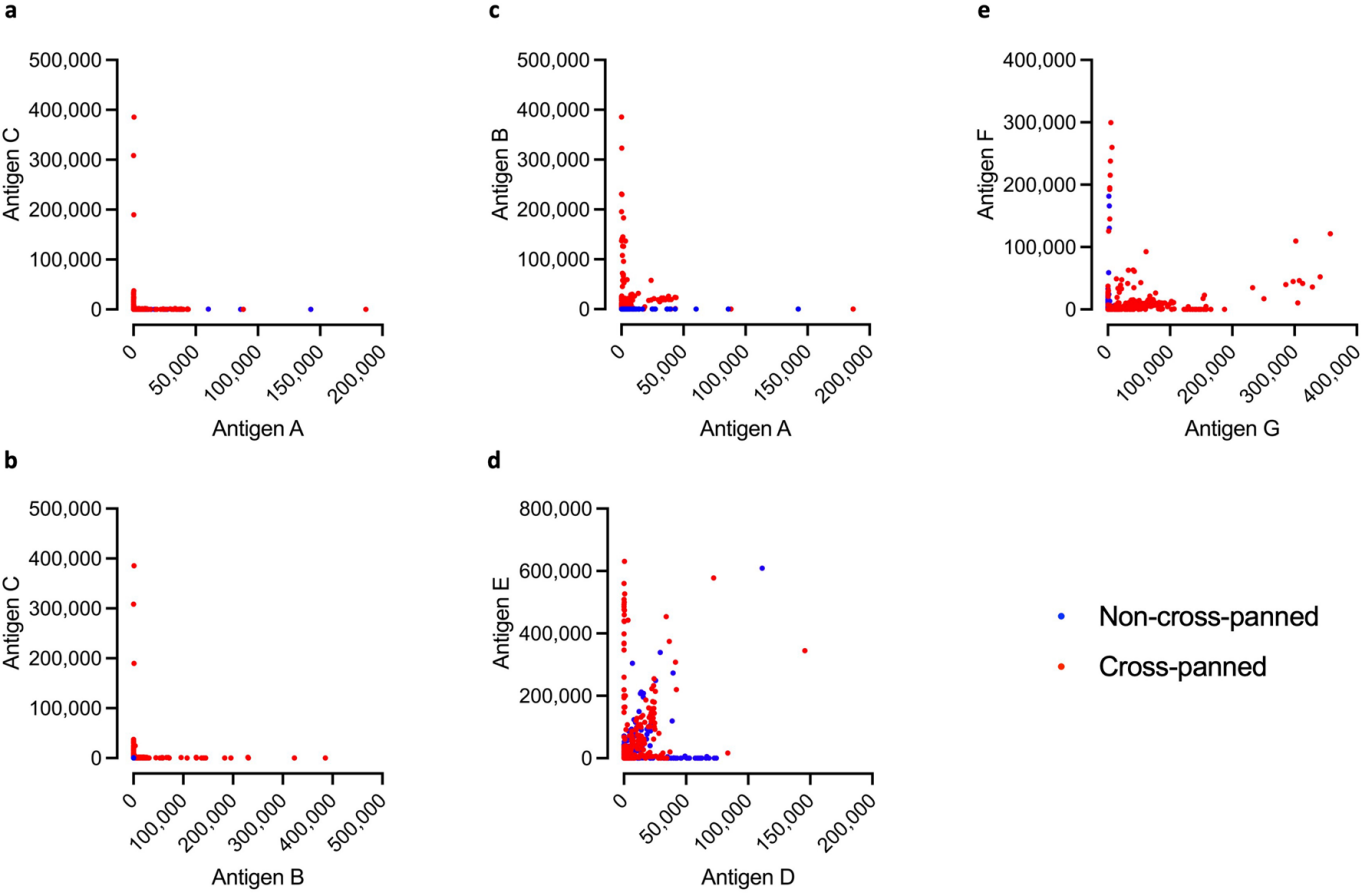
Cross-reactivity of scFvs in ENC DELFIAs. On the graphs, the binding signal (DELFIA TRF 320 nm/615 nm measured on a Victor Nivo Multimode Microplate Reader) to one antigen is shown on the X-axis, and to a second antigen on the Y-axis. Binding signals are calculated as raw signal output, minus the values for negative controls on respective assay plate. Negative values have been set to 0. The graphs include the results from 753, 978, and 644 tested scFvs, respectively from the PLA_2_, LNTX, and SNTX panning campaigns. “Non-cross-panned” refers to panning rounds (Fig. 1b and Supplementary Fig. S1) including only letters of one type (AA-, AAA, DD-, DDD, etc.), whereas “Cross-panned” refers to panning rounds with different letters (AB-, AAB, DE-, DEE, etc.). In the ENC DELFIA, anti-FLAG antibodies are coated to the plate and bind the bacterially expressed scFvs via their FLAG-tag. Thereby, the amount of coated anti-FLAG antibodies facilitate that the same amount of scFvs (regardless of expression level, specificity, and sequence) is captured in each well (assuming saturation in the capture step), which results in the experiment being less biased by different scFv expression levels.

**Figure 4:**
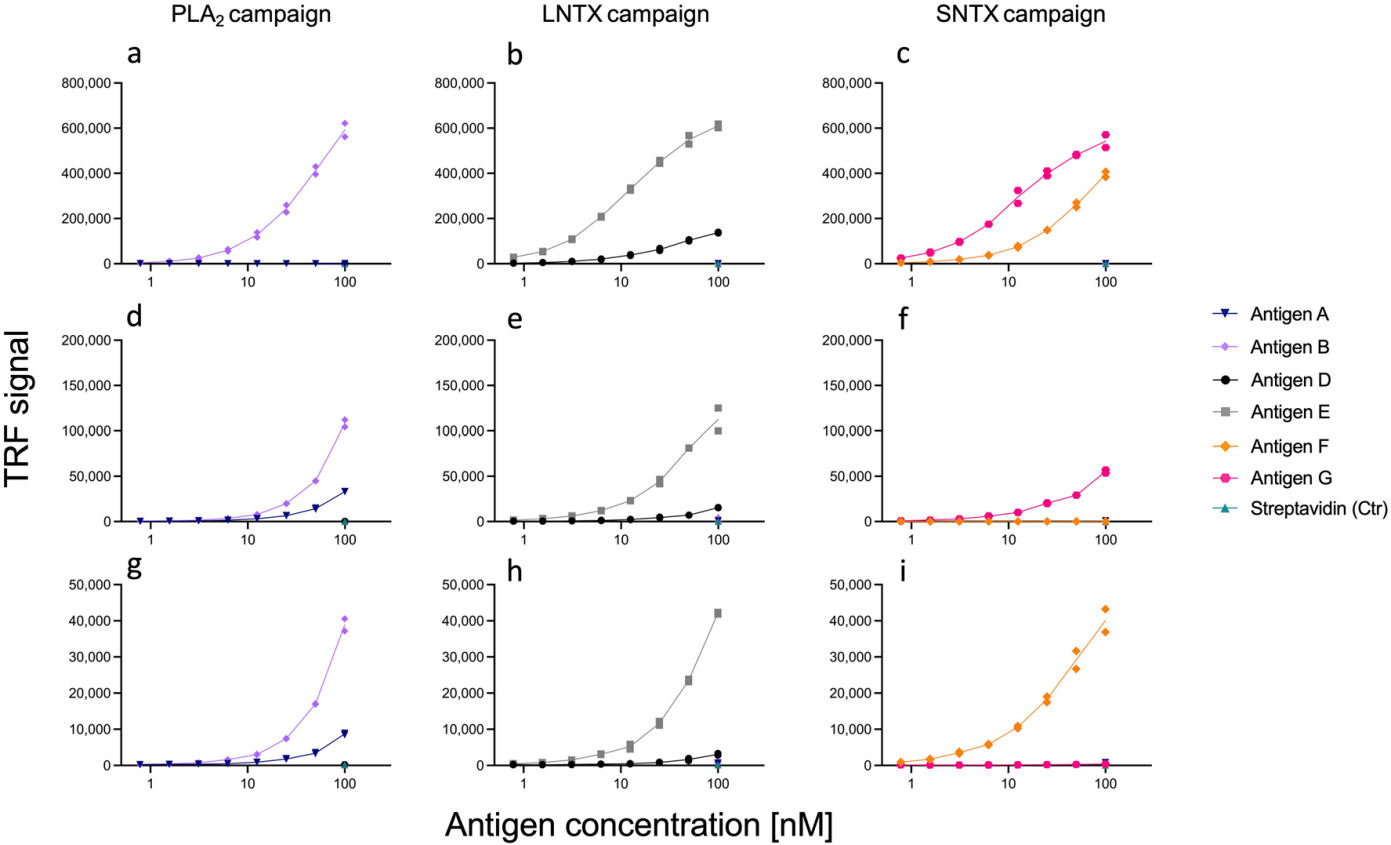
Titration DELFIAs. This figure shows examples of titration DELFIA graphs for three scFvs from each of the PLA_2_, LNTX, and SNTX campaigns. In Supplementary Fig. S2 the titration DELFIAs for all tested scFvs can be observed. Three control antigens, streptavidin and two of the antigens not used in the campaign for the discovery of the given scFv (i.e., antigens used in the SNTX campaign were used as control antigens in the PLA_2_ campaign, etc.), were included at the highest concentration (100 nM). In the following descriptions, (CP) and (NCP) refers to antibodies originating from cross-panned or non-cross-panned selectings respectively. (**a**) TPL0127_02_A02 (CP), (**b**) TPL_0067_02_E08 (NCP), (**c**) TPL0229_02_A03 (CP), (**d**) TPL0127_02_H06 (CP), (**e**) TPL0066_01_B08 (NCP), (**f**) TPL0230_02_F07 (CP), (**g**) TPL0127_01_F09 (CP), (**h**) TPL0065_02_E02 (CP), and (**i**) TPL0227_01_E01 (NCP).

**Figure 5:**
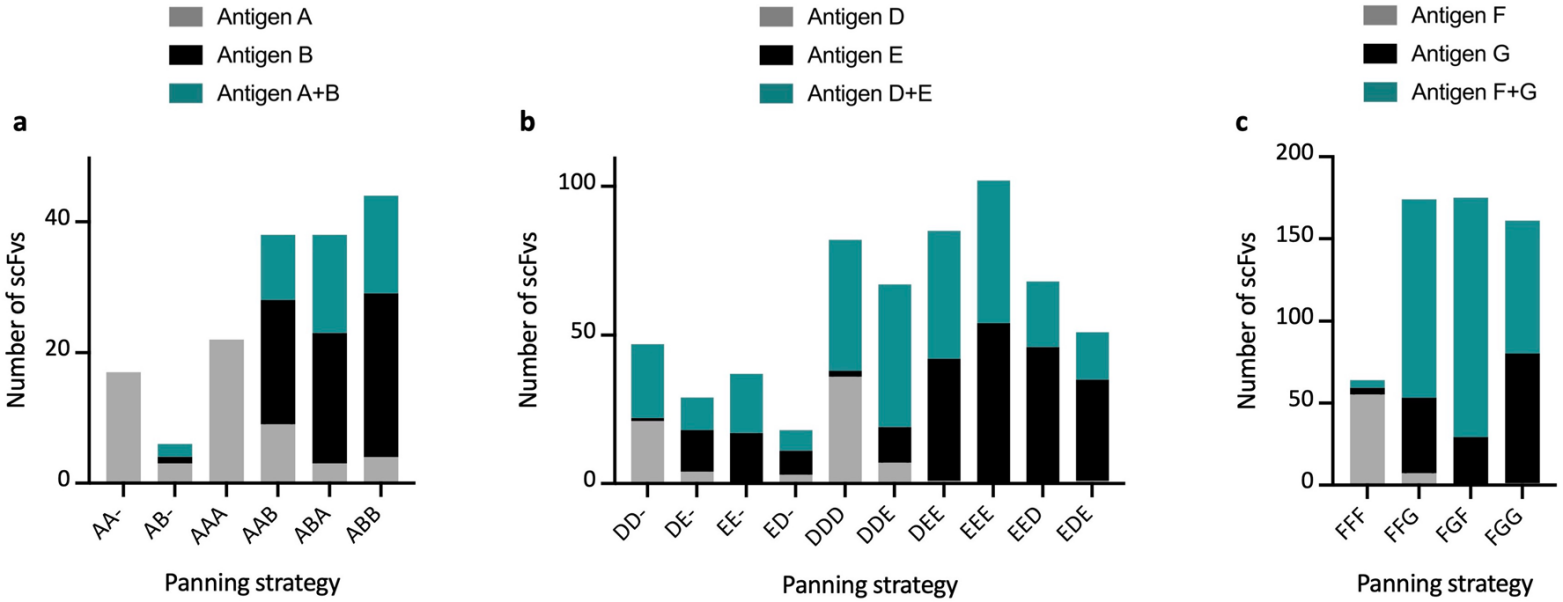
Comparison of panning strategies. This figure shows the number of scFvs with positive binding signals divided into the different panning strategies. A positive binding signal is determined as a signal value above 2000 after the negative controls have been subtracted. Monoclonal scFvs able to bind to both antigens were only reported as cross-binders and removed from the individual antigen signals. i.e. an scFv binding to antigens A and B was registered as binding to antigen A + B, and not as binding to antigen A and antigen B as well. Panning strategies ending with a hyphen “-“ refers to the second round of panning.

Evaluating the monoclonal scFvs through expression normalized capture (ENC) DELFIAs, it was observed that cross-panning tends to enhance the chance of identifying cross-reactive scFvs (Fig. 3). However, an exception to this trend was seen in the LNTX campaign (Fig. 3d), where high levels of cross-reactivity were also detected in scFvs resulting from non-cross-panned strategies. Comparing across campaigns, the LNTX and SNTX campaigns demonstrated higher cross-reactive scFv signals in the ENC DELFIAs than the PLA_2_ campaign when compared with the non-cross-reactive scFvs. This could indicate that selecting for cross-reactivity is particularly challenging when working with the PLA_2_s. The monoclonal scFv ENC DELFIAs further revealed that we were unable to discover cross-reactive scFvs against PLA_2_s when antigen C was included in the cross-panning strategies (Fig. 3a and b). Therefore, antigen C was excluded in the subsequent cross-reactivity analysis. Titration DELFIAs were carried out to ensure that the scFvs bind specifically and that cross-binding was not a result of non-specific binding (i.e., polyreactivity). These experiments showed that the tested subset of scFvs bind specifically to their cognate antigens (Fig. 4 and Supplementary Fig. S2). From these binding data, no clear trend seems to emerge regarding which cross-panning strategy yielded the highest number of cross-reactive scFvs (Fig. 5).

### Many unique scFv sequences share the same HCDR3

**Figure 6:**
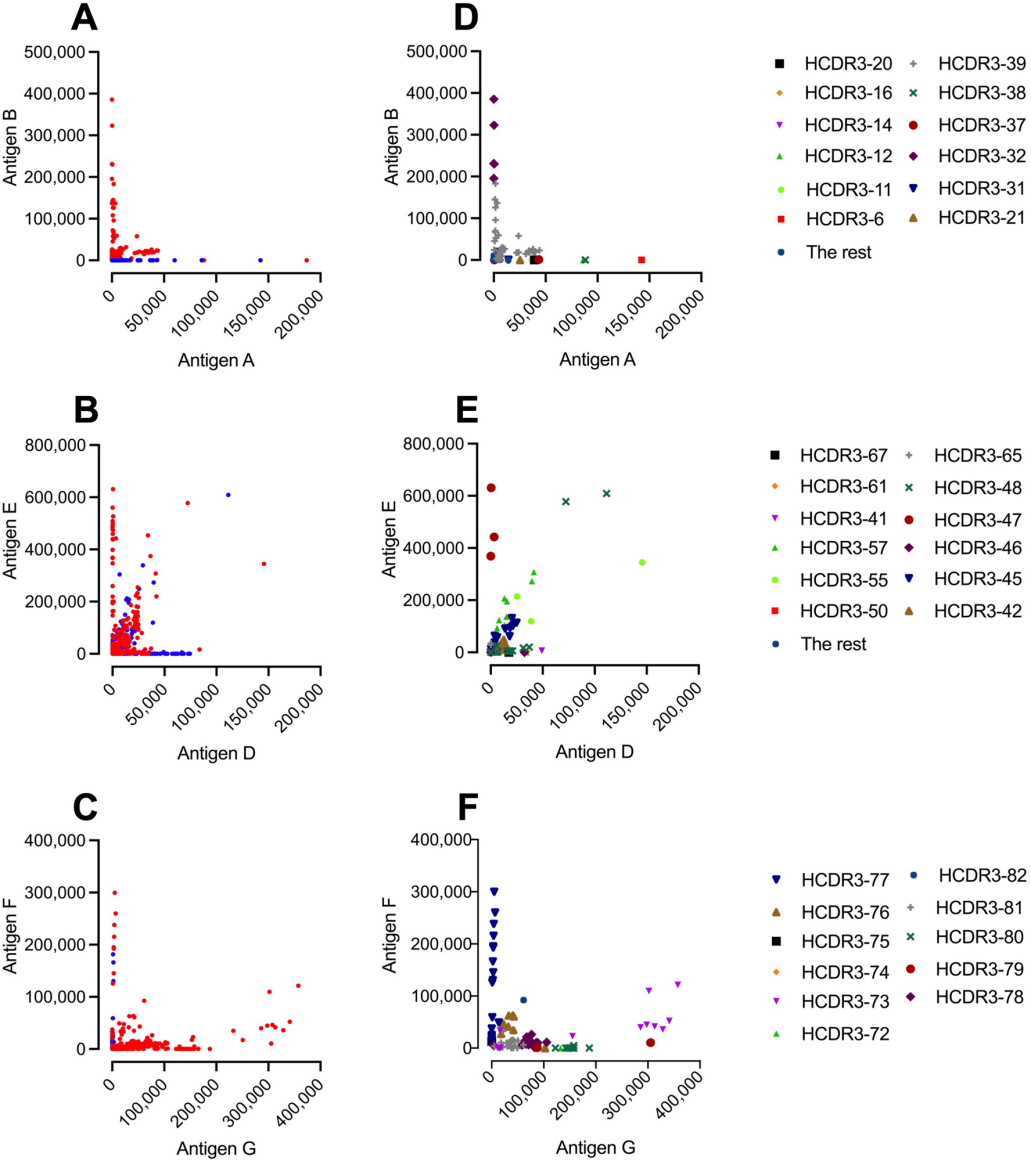
Overview of unique HCDR3 regions. Panel (**A**, **B**, and **C**) are identical to panel (**C**, **D**, and **E**) in Fig. 3, and show binding to antigens, and whether cross-panning strategies (Red) were used for discovery, or not (Blue). Panel (**D**, **E**, and **F**) show the scFvs that were sequenced, and which of the scFvs have identical HCDR3 regions. In the legend on the right side, the different unique HCDR3 are labeled. To simplify the graph, the less prevalent HCDR3 families have been grouped in the “The rest” category.

To confirm that we were observing different scFvs and not merely analyzing a few unique scFvs, we carried out Sanger sequencing of selected scFvs. The sequencing results were as follows: i) Of 101 sequenced scFvs from the PLA_2_ campaign, 72 unique scFvs were discovered, containing 39 unique heavy chain complementarity determining region 3 (HCDR3); ii) Of 109 sequences from the LNTX campaign, 64 unique sequences were discovered, containing 32 unique HCDR3; iii) Of 123 sequences from the SNTX campaign, 39 unique sequences were discovered, containing 11 unique HCDR3. From this initial sequence analysis, it was evident that we had indeed identified a wide variety of unique scFvs. However, we further wanted to visualize the binding of scFvs with different HCDR3s by combining these sequencing results with the previously obtained binding data for the scFvs (Fig. 6).

The overlay allows for several observations (Fig. 6). Firstly, all the scFvs showing cross-reactivity to antigen A and B share the same HCDR3 (Fig. 6D). However, this HCDR3 (HCDR3-39) is not exclusive to cross-reactive scFvs, as it also appears in scFvs that bind solely to antigen B. Further, despite the occurrence of HCDR3-39 in 38 sequences, 22 of these sequences are actually unique (Table 2). Secondly, in the LNTX campaign (Fig. 6E), HCDR3-48 appears in 15 scFvs, 9 of which are unique (Table 2). However, only 2 of these 15 scFvs demonstrate high binding signals, whereas the other 13 show low binding signals. Lastly, the cluster of scFvs containing HCDR3-73 is the HCDR3 cluster that shows the highest cross-reactive binding in the SNTX campaign to antigens F and G (Fig. 6F). However, scFvs featuring this HCDR3 also show medium binding to only antigen G or no binding to any of the antigens. scFvs with identical HCDR3s but differing light-chain pairings generally presented different binding profiles in the titration DELFIAs (Supplementary Fig. S2 panel n and y compared to w and ac respectively), indicating that the light-chains may play a role in the (cross-)binding properties of the scFvs.

**Table 2:** Overview of the three most common HCDR3s of each campaign. The table shows the three most prevalent HCDR3 for each campaign, and how many unique scFv sequences contain identical HCDR3 regions.

Campaign	scFvs containing this HCDR3 region	Unique scFvs containing this HCDR3 region
PLA_2_ campaign		
HCDR3-39	38	22
HCDR3-27	10	2
HCDR3-24	6	2
LNTX campaign		
HCDR3-48	15	9
HCDR3-56	12	7
HCDR3-42	12	2
SNTX campaign		
HCDR3-78	30	7
HCDR3-77	22	6
HCDR3-81	20	7

## Discussion

In this study, we conducted numerous antibody phage display selection campaigns using a naïve scFv antibody library and cross-panning against snake toxins from three different protein (sub)families, with the aim of exploring determinants driving the selection of cross-reactive antibodies. By analyzing the antigen binding of the monoclonal scFvs discovered from 25 phage display selection campaign outputs, we observed an increased (or equal) accumulation of cross-reactive scFvs when panning involved alternating antigens in consecutive selection rounds, compared to when only the same antigen was used (Fig. 5). However, when the alternating antigens were highly dissimilar (Table 1: Antigen C compared to A and B), we were unable to identify cross-reactive scFvs, as seen with antigen C among the PLA_2_s (Fig. 3a and b).

For the LNTX campaign, a notable observation was that both selection strategies (including cross-panning or not) resulted in the discovery of cross-reactive scFvs (Fig. 3d). Conversely, cross-panning appeared to be necessary for the discovery of cross-reactive scFvs targeting PLA_2_s and SNTXs, respectively (Fig. 3c and e). To understand this difference, we investigated three parameters in the antigen analysis: sequence, structure, and surface similarity (Table 1 and Fig. 2). While the LNTXs neither shared the highest sequence similarity nor the highest surface conservation, they did show the highest RMSD score. However, a high RMSD score refers to a high deviation between antigen structures (less similarity) and is therefore likely not the explanation for why cross-panning was not necessary, although it cannot be completely dismissed as a potential confounding factor.

Another notable observation is that the PLA_2_-binding cross-reactive scFvs show lower ENC DELFIA binding signals (indicating lower affinity to the target) than the non-cross-reactive PLA_2_-binding scFvs (Figs. 3c, 4), which is generally not observed for the scFvs from the LNTX or SNTX campaigns (Figs. 3d, e and 4). The PLA_2_s had a pruned RMSD score in between the scores for the LNTXs and SNTXs and a surface conservation that was very similar to the LNTXs. Neither of these factors explains why the PLA_2_s should be more difficult to discover high-affinity cross-reactive scFvs against (Table 1 and Fig. 2). The PLA_2_s did, however, show the lowest linear sequence similarity (58%), but since this was not much lower than for the LNTXs (64% sequence similarity), we do not expect this to be the sole explanation for the difficulty in obtaining high-affinity PLA_2_-binding cross-reactive scFvs. Taken together, the antigen analysis did not provide definitive answers for either of the observed discrepancies, but rather highlighted the difficulty in predicting cross-reactivity based on global similarity analysis.

Earlier studies aiming to discover cross-reactive antibodies using phage display cross-panning strategies mention that epitope similarity and antigen function are important to take into consideration when planning the discovery of cross-reactive antibodies^3–5,8^. Bearing this in mind, we compared the antigen function of our included antigens. The primary functions of the PLA_2_ antigens A, B, and C differ in the sense that antigen C is an Asp49 PLA_2_, with the main function of being able to hydrolyze the sn-2 position of the glycerol backbone in phospholipids. In contrast, antigen A and B are Ser49 and Lys49 PLA_2_s, respectively, and are devoid of enzymatic activities, and function by destabilizing cell membranes in other ways^16,17^. Antigen A has been reported to be quite different in structure when complexed with either lauric acid or suramin compared to Lys49 PLA_2_-like toxins, such as antigen B^17^, which may indicate that these antigens differ functionally. In contrast, both LNTXs non-enzymatically target the nicotinic acetylcholine receptor (nAChR) and share similar binding activity profiles^4,5,18^. Likewise, the SNTXs have also been reported to target the nAChR with comparable affinities and exert similar toxic effects^19^. Therefore, it seems plausible that both the LNTXs and SNTXs share structurally similar motifs related to their function, which could potentially explain why some antigens are more feasible to discover high-affinity cross-reactive scFvs against than others. In the future, we hope more epitope information becomes available for snake toxins to facilitate the analysis of this characteristic.

## Conclusion

In summary, this exploratory study helps shed light on the determinants driving the discovery of cross-reactive antibodies using phage display technology. It offers the first broad demonstration of how cross-panning can be used to increase the fraction of cross-reactive antibodies in the panning outputs from phage display selection campaigns. Several discrepancies were observed regarding the feasibility of discovering cross-reactive antibodies against certain antigens. Linear, structural, and surface similarity of the antigens did not account for these differences. However, when antigens with the same function (and thereby sharing similar structural motifs in their functional sites) were used as alternating targets during cross-panning, our observations indicate that it might be more feasible to select high-affinity cross-reactive antibodies, than when other antigens with dissimilar functions are used as alternating targets. Taken together, this study demonstrates the difficulty in predicting how antibody cross-reactivity can be rationally selected for a priori using phage display technology, but that cross-panning may often be beneficial. Understanding the drivers behind antibody cross-reactivity would not only be important for the discovery of cross-reactive antibodies, but could also, conversely, facilitate the discovery of highly specific non-cross-reactive antibodies. Finally, fields like vaccine development, antibody-based diagnostics, and the development of antibodies with broad toxin-neutralizing capabilities against infectious diseases and animal envenomings stand to benefit from a deeper understanding of antibody cross-reactivity. We hope that the findings presented here may aid research and development efforts in these areas.

## Materials and methods

### Linear similarity

To investigate the linear similarity of the antigens, linear alignment was carried out using CLC Main Workbench 21.0.4, with gap open cost and gap extension cost of 10. Correct alignment was quantified manually, and an identity percentage was calculated based on this. Antigen sequences were retrieved from Uniprot.org under the following Uniprot IDs: Ecarpholin S (P48650), myotoxin II (P24605), Acidic PLA_2_ 2 (P15445), α-cobratoxin (P01391), α-elapitoxin (P01397), short neurotoxin 1 (P01426), and erabutoxin A (P60775). For antigens with signal peptides (myotoxin II and erabutoxin A) included in the Uniprot residue sequence, the signal peptides were removed.

### Structural comparison through superimposing

To investigate the structural similarity of the antigens, the respective structures for ecarpholin S (P48650; 2QHD), myotoxin II (P24605; 1CLP), acidic PLA_2_ 2 (P15445; 1A3D), α-cobratoxin (P01391; 1CTX), α-elapitoxin (P01397; AF-P01397), short neurotoxin 1 (P01426; 1IQ9), and erabutoxin A (P60775; 1QKD) were retrieved. They were imported into ChimeraX^20^ and superimposed via the matchmaker module, which superimposed the toxin structures by first creating a pairwise sequence alignment and then fitting the aligned residue pairs. Root-mean-square deviation (RMSD) was calculated across the whole protein alignment, as well as only for the sections successfully superimposed (pruned atom-pair distance < 2 Å) to also assess similarities in the absence of major atom-pair outliers. Based on the alignment, the conservation values were calculated with the entropy-based measure from AL2CO^21^ and surface conservation was visualized with maroon indicating high (0.9) and blue low conservation (-1.3); white represented elements present in one, but absent in the other (-0.2).

### Antigen procurement

Short neurotoxin 1 (L8101), erabutoxin A (L8110), α-cobratoxin (L8114), and whole venoms from *Naja naja* (L1324), *Echis carinatus sochureki* (L1111), and *Dendroaspis polylepis* (L1309) were purchased from Latoxan S.A.S., France. Myotoxin II (P24605) was purified from whole *Bothrops asper* venom using cation-exchange chromatography on CM-Sephadex C25, followed by reverse phase HPLC on C_18_ as described elsewhere^22,23^. Venom fractions containing the toxins of interest (Nn9 containing acidic phospholipase A_2_ 2 (P15445) from *N. naja*, Ecs13 and Ecs14 containing ecarpholin S (P48650) from *E. c. sochureki*, and Dp7 containing α-elapitoxin-Dpp2c^24^ (P01397) from *D. polylepis*) were separated using RP-HPLC (Agilent 1200) as described elsewhere^4^. The fraction Dp7 was described elsewhere to contain α-elapitoxin-Dpp2c (P01397), however, the fraction composition of the *N. naja* and *E. c. sochureki* chromatograms were undescribed. To investigate which fractions contained the toxins of interest in these two venoms, the fractions were analyzed by Proteomics Core at the Technical University of Denmark for LC–MS/MS. The LC–MS/MS, the following analysis of the resulting peptide spectra, and the quantification using label-free quantification (LFQ) were carried out as described elsewhere^25^. The chromatograms and LFQ results are shown in Supplementary Figs. S3 and S4. Following fractionation, samples were evaporated using a vacuum centrifuge and resuspended in PBS.

### Biotinylation of antigens

Antigens were biotinylated as described elsewhere^26^, using the following molar ratios (toxin:biotin). For ecarpholin S, myotoxin II, and acidic PLA_2_ 2 a ratio of 1:1.5 was used. For short neurotoxin 1 and erabutoxin A, ratios of 1:1.5 and 1:1.25 were used, respectively. Lastly, for α-cobratoxin and α-elapitoxin-Dpp2c a ratio of 1:1.5 was employed. Following biotinylation and purification, an analysis of the level of biotinylation was carried out as described earlier^26^.

### Antibody phage display selection

The naïve IONTAS human scFv-based antibody phage display library^27^, containing a clonal diversity of 4 × 10^10^, was employed for phage display selection. The IONTAS library was created with the variable heavy and light chain antibody genes from the naïve IgM repertoire from 43 healthy human donors^27^. Phage display selection campaigns were carried out using two different antigen-immobilization techniques. For the PLA_2_s and LNTXs, biotinylated antigens were immobilized in streptavidin-coated MaxiSorp vials using 10 µg/mL antigen concentration. Phage display selection was carried out as described elsewhere^27^, with the same protocol modifications as described by Ahmadi et al.^9^*.* For the SNTXs, biotinylated antigens were immobilized on streptavidin-coated dynabeads using 0.7 µg/mL antigen and phage display selection was carried out as described elsewhere^27^, with the same protocol modifications as described by Ledsgaard et al. ^4^. An overview of the cross-panning strategies can be seen in Fig. 1b.

### Polyclonal phage ELISA and subcloning

Following three rounds of selection, the phage outputs were screened for the polyclonal binding properties, using an ELISA protocol adapted from Pershad et al.^28^. The assays were carried out in clear 96-well MaxiSorp plates (Nunc) using 10 µg/mL overnight (4 °C) streptavidin or neutravidin coat. Biotinylated antigens were added at a concentration of 5 µg/mL in 3% MPBS, followed by addition of the respective phage outputs. For detection, a 1:2,000 dilution of anti-M13-HRP antibody (Sino Biopharmaceuticals) in 3% MPBS was used combined with TMB substrate solution (ThermoFisher Scientific). The HRP/TMB reaction was stopped using 1 M sulfuric acid and absorption was measured at 450 nm using a Victor Nivo Multimode Microplate Reader (PerkinElmer). Upon successful primary screening, the scFv-encoding genes were subcloned from the pIONTAS1 phagemid vector into the pSANG10-3F expression vector as described elsewhere^26^.

### ENC DELFIAs and Sanger sequencing

Black MaxiSorp plates (Nunc) were coated overnight with anti-FLAG M2 antibody (Sigma, 2.5 ug/mL in PBS) at 4 °C. After blocking, individual autoinduction supernatants^29^ containing monoclonal FLAG-tagged scFvs in 3% MPBS were added. Thereafter, antigens were added at 25 nM for SNTX and 100 nM for LNTX and PLA_2_s in the one-dose experiment, and at a concentration range between 0.78 nM and 100 nM for the titration DELFIA. Binding was detected using europium labeled streptavidin (PerkinElmer 1244–360, 200 ng/mL) in DELFIA assay buffer (PerkinElmer 4002–0010), and DELFIA enhancement solution (PerkinElmer​ 4001–0010). Binding was measured as a TRF signal at 320 nm excitation and 615 nm emission. Following ENC DELFIAs, 333 scFvs were cherry-picked and sequenced (Eurofins genomics sequencing service) using the T7 Eurofins standard primer (TAATACGACTCACTATAGGG).

## Supplementary Information

Supplementary Information 1.

Supplementary Information 2.

Supplementary Information 3.

Supplementary Information 4.

Supplementary Information 5.

Supplementary Information 6.

## Data Availability

The datasets used and/or analysed in the current study are available from the corresponding author on reasonable request. Antibody sequences will be made available on request after completion of a Material Transfer Agreement.
