# Supplementary material for: Cross-reactivity trends when selecting scFv antibodies against snake toxins using a phage display-based cross-panning strategy
Source: Sci Rep. 2023 Jun 22;13:10181. doi: 10.1038/s41598-023-37056-6 (PMC10287648; doi:10.1038/s41598-023-37056-6)
Supplement: Supplementary file 1 — Supplementary Information 1. [file 41598_2023_37056_MOESM1_ESM.docx]

***Supplementary Figure S1.*** ***Polyclonal phage ELISA signals for the phage outputs of each selection strategy from selection round 2 and 3****. (****a****) PLA_2_ campaign, (****b****) LNTX campaign, and (****c****)* *SNTX campaign.* *Measurements were carried out in duplicates or triplicates with standard deviation depicted as error bars. Panning strategies with two letters and a hyphen (e.g., AA-) correspond to a second-round phage output, whereas three letters (e.g., AAA) refer to a third-round output. Streptavidin, neutravidin, and milk proteins are included as negative controls.*

***Supplementary Figure S2. Titration DELFIA graphs for 12 scFvs from each of the PLA_2_, LNTX, and SNTX campaigns****. Three control antigens, streptavidin and two of the antigens not used in the campaign for the discovery of the given scFv (i.e., antigens used in the SNTX campaign were used as control antigens in the PLA_2_ campaign, etc.), were included at the highest concentration (100 nM). In the following descriptions, (CP) and (NCP) refer to antibodies originating from cross-panned or non-cross-panned selections, respectively. The individual scFvs can be mapped back to the XY plots in Fig. 3 using Supplementary Fig. S3 and the following antibody labels (****a****) TPL0123_02_A06 (NCP), (****b****) TPL0127_02_A02 (CP), (****c****) TPL0127_02_E05 (CP), (****d****) TPL0126_02_A03 (CP), (****e****) TPL0124_02_C06 (CP), (****f****) TPL0127_01_F09 (CP), (****g****) TPL0127_02_A06 (CP), (****h****) TPL0127_01_A07 (CP), (****i****) TPL0127_02_H06 (CP), (****j****) TPL0124_02_F05 (CP), (****k****) TPL0124_02_B08 (CP), (****l****) TPL0126_02_A06 (CP), (****m****) TPL0101_01_E10 (CP), (****n****) TPL0067_02_E08 (NCP), (****o****) TPL0066_01_D05 (NCP), (****p****) TPL0065_02_C03 (CP), (****q****) TPL0067_01_G09 (NCP), (****r****) TPL0067_02_A07 (NCP), (****s****) TPL0067_01_C03 (NCP), (****t****) TPL0097_02_B08 (NCP), (****u****) TPL0066_01_A02 (NCP), (****v****) TPL0066_01_B08 (NCP), (****w****) TPL0067_01_E01 (NCP), (****x****) TPL0065_02_E02 (CP), (****y****) TPL229_01_G09 (CP), (****z****) TPL0228_01_A08 (CP), (****aa****) TPL0229_02_F06 (CP), (****ab****) TPL0228_01_C10 (CP), (****ac****) TPL0230_02_F07 (CP), (****ad****) TPL0227_01_E01 (NCP), (****ae****) TPL0229_02_A03 (CP), (****af****) TPL0229_02_A02 (CP), (****ag****) TPL0228_01_C10 (CP), (****ah****) TPL0228_02_G03 (CP), (****ai****) TPL0228_01_A03 (CP), and (****aj****) TPL0230_01_B01 (CP).*

***Supplementary Figure S3. Overview of scFvs picked for titration DELFIAs.*** *This figure maps back the scFvs tested in the titration DELFIAs to the ENC DELFIAs in Fig. 3. (****a****) scFvs from the PLA_2_s campaign, (****b****) scFvs from the LNTX campaign, and (****c****) scFvs from the SNTX campaign*

***Supplementary Figure S4. Echis carinatus sochureki fractionation and proteomic analysis.*** *(****a****) RP-HPLC chromatogram, with collected fractions labelled. (****b****) Fraction composition determined by LC-MS/MS and quantified by LFQ. Each fraction is labeled with species acronym (Ecs) followed by fraction number, relating to the fractions in Figure* ***a****.*

***Supplementary Figure S5. Naja naja fractionation and proteomic analysis.*** *(****a****) RP-HPLC chromatogram, with collected fractions labelled. (****b****) Fraction composition determined by LC-MS/MS and quantified by LFQ. Each fraction is labeled with species acronym (Nn) followed by fraction number, relating to the fractions in Figure* ***a****.*
