# Supplementary figures and images for: Cross-reactivity trends when selecting scFv antibodies against snake toxins using a phage display-based cross-panning strategy
Source: Sci Rep. 2023 Jun 22;13:10181. doi: 10.1038/s41598-023-37056-6 (PMC10287648; doi:10.1038/s41598-023-37056-6)

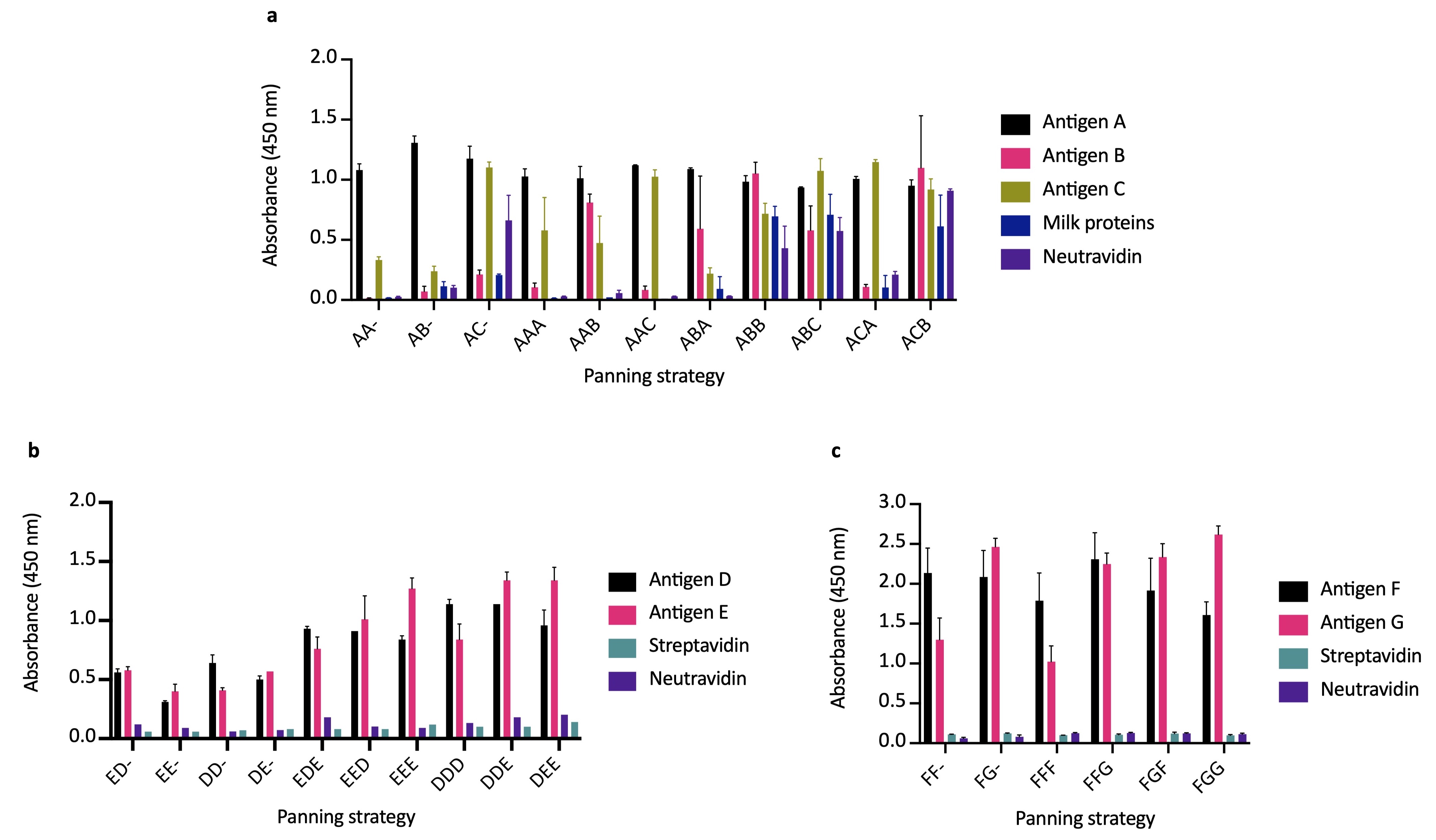

Supplement: Supplementary file 2 — Supplementary Information 2. [file 41598_2023_37056_MOESM2_ESM.jpg]

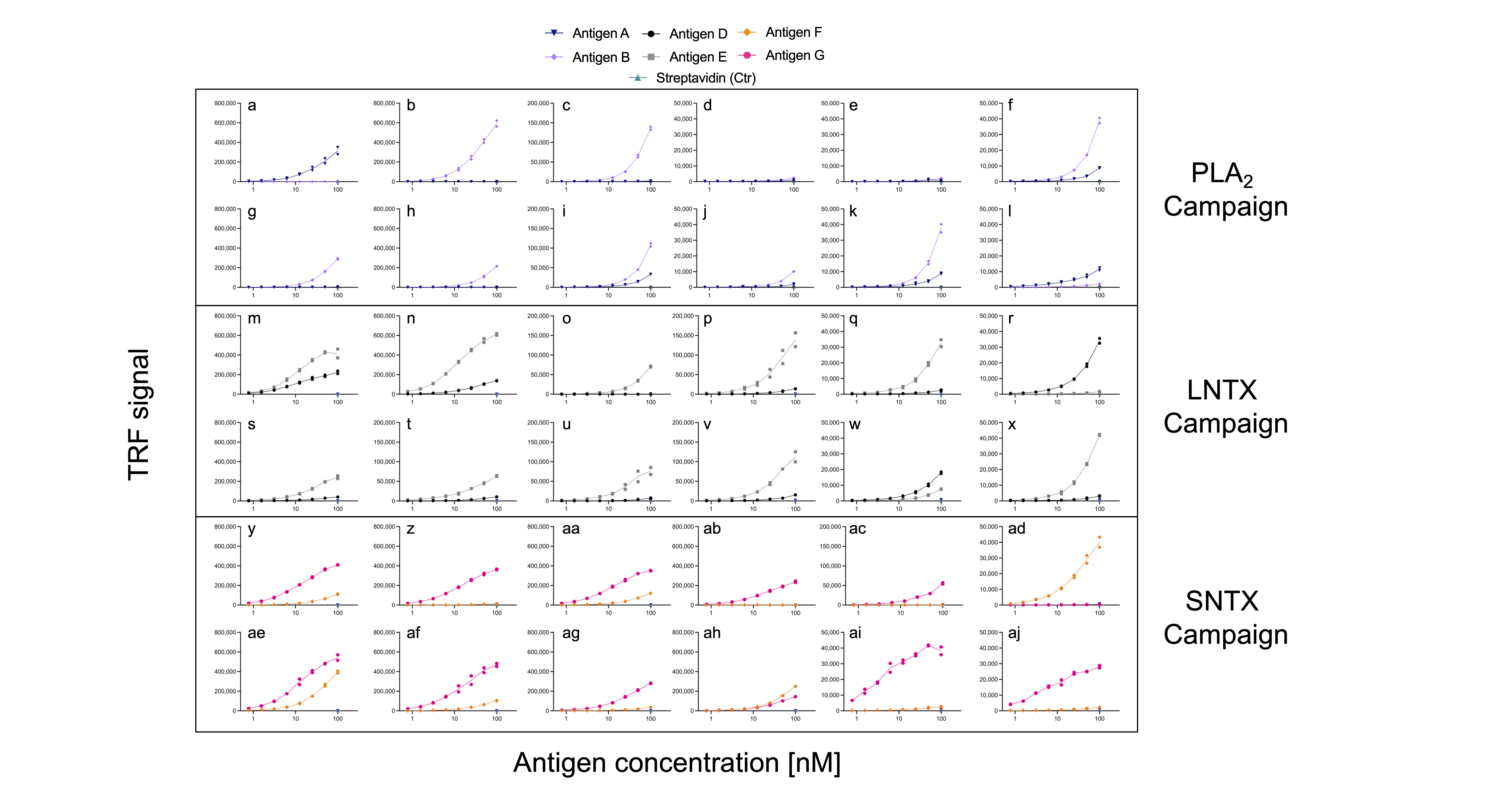

Supplement: Supplementary file 3 — Supplementary Information 3. [file 41598_2023_37056_MOESM3_ESM.jpg]

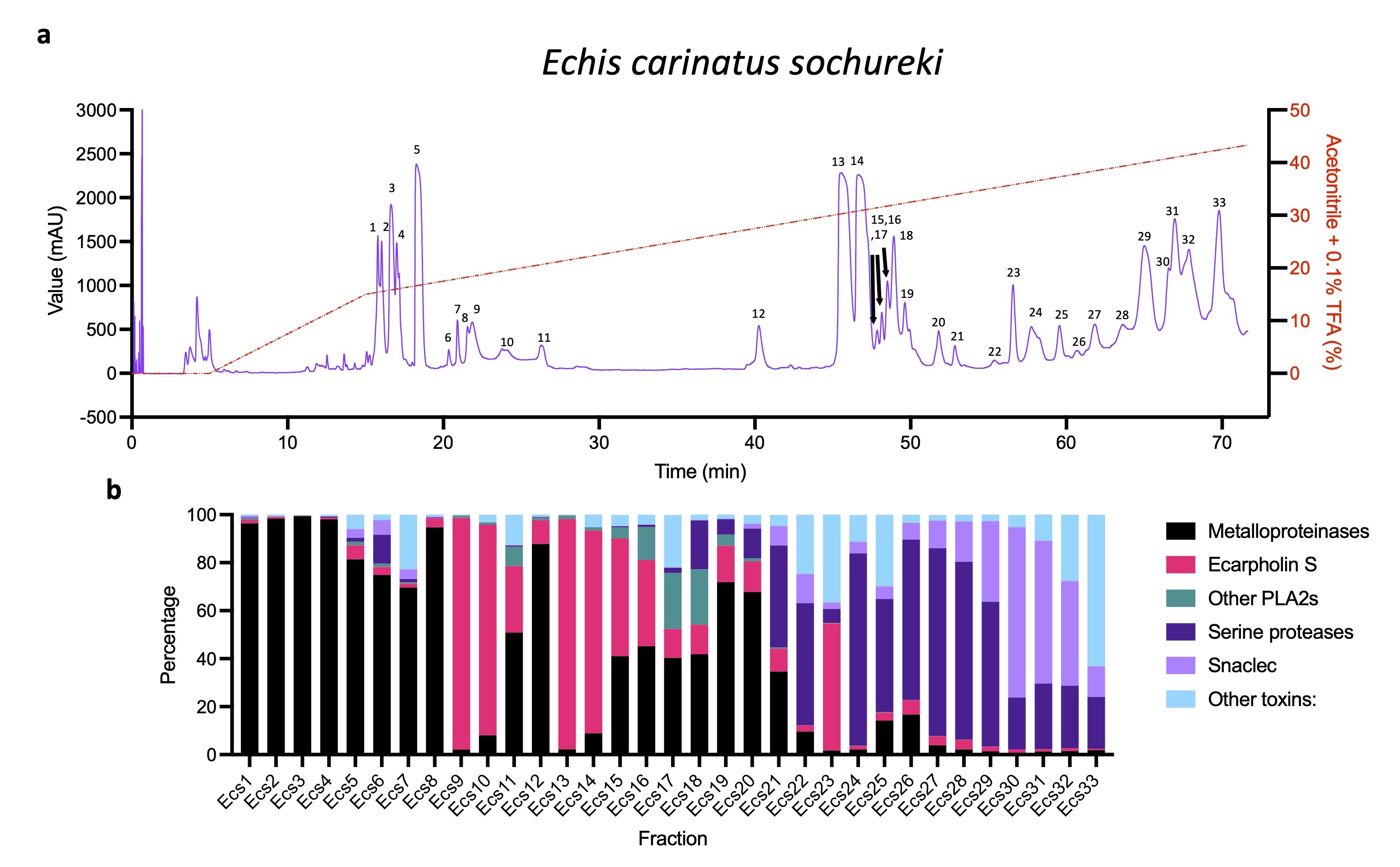

Supplement: Supplementary file 5 — Supplementary Information 5. [file 41598_2023_37056_MOESM5_ESM.jpg]

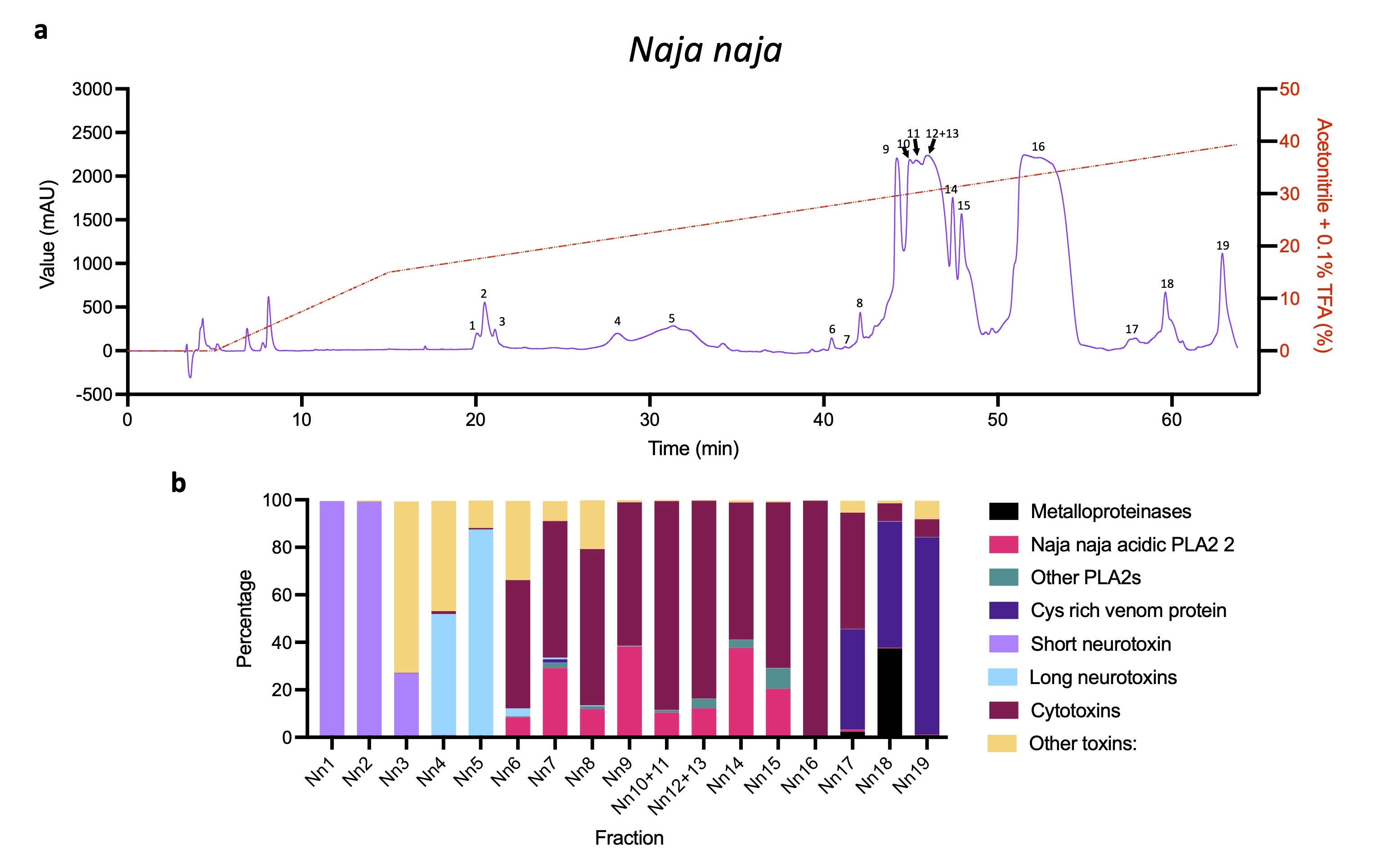

Supplement: Supplementary file 6 — Supplementary Information 6. [file 41598_2023_37056_MOESM6_ESM.jpg]
